# Supplementary material for: Selective Photocatalytic Transformation of Lignin to Aromatic Chemicals by Crystalline Carbon Nitride in Water–Acetonitrile Solutions
Source: Int J Environ Res Public Health. 2022 Nov 25;19(23):15707. doi: 10.3390/ijerph192315707 (PMC9736535; doi:10.3390/ijerph192315707)
Supplement: Supplementary file 1 [file ijerph-19-15707-s001.zip › ijerph-2053710-supplementary.pdf]

International Journal of
Environmental Research and Public Health

MDPI

Supplementary Information

Selective Photocatalytic Transformation of Lignin to Aromatic Chemicals by Crystalline Carbon Nitride in Water–Acetonitrile Solutions

Meirou Huang [1,2], Huiqin Guo [1,2,*], Zhenxing Zeng [3,*], Hong Xiao [3], Hong Hu [1,2], Liu He [1,2], Kexin Li [1,2], Xiaoming Liu [1,2] and Liushui Yan [1,2,*]

1 Key Laboratory of Jiangxi Province for Persistent Pollutants Control and Resources Recycle, Nanchang Hangkong University, Nanchang 330063, China
2 Jiangxi Provincial Experimental Teaching Demonstration Center of Environmental Science and Engineering, Nanchang Hangkong University, Nanchang 330063, China
3 College of Environmental Science, Sichuan Agricultural University, Chengdu 611130, China
* Correspondence: guohuiqin@nchu.edu.cn (H.G.); zzx207@hotmail.com (Z.Z.); yanliushui@nchu.edu.cn (L.Y.)

Citation: Huang, M.; Guo, H.; Zeng, Z.; Xiao, H.; Hu, H.; He, L.; Liu, X.; Yan, L. Selective Photocatalytic Transformation of Lignin to Aromatic Chemicals by Crystalline Carbon Nitride in Water–Acetonitrile Solutions. *Int. J. Environ. Res. Public Health* **2022**, *19*, x. https://doi.org/10.3390/ijerph192315707

Academic Editor: Paul B. Tchounwou

Received: 9 November 2022
Accepted: 23 November 2022
Published: 25 November 2022

Publisher's Note: MDPI stays neutral with regard to jurisdictional claims in published maps and institutional affiliations.

Copyright: © 2022 by the authors. Submitted for possible open access publication under the terms and conditions of the Creative Commons Attribution (CC BY) license (https://creativecommons.org/licenses/by/4.0/).

Supplementary Text

1. Analysis Methods

The lignin model compound (2-phenoxy-1-phenylethanol) and the resulting product after its conversion were qualitatively analyzed by GC-MS and quantitatively analyzed by HPLC (external standard method). The specific detection conditions of GC-MS are as follows: DB-17MS column (30 m × 0.25 mm × 0.25 μm), detector temperature is 280 °C, the injection volume is 1.0 μL, injection port temperature is 250 °C. High purity He is used as the carrier gas and the flow rate is 1.0 mL/min, without splitting. Heating procedure: initial temperature 50 °C, hold for 3 min; then the temperature was raised at 10 °C/min for 2 min, maintained for 5 min; after that, raising at a warming rate of 2 °C/min for 5 minutes, maintained for 2 minutes; finally, raising at a temperature rate of 15 °C/min for 12 minutes and maintained for 2 minutes. The specific detection conditions for HPLC are as follows: the injection volume is 10 μL; the mobile phase was a mixture of water(A) and acetonitrile(B); the gradient elution was as follows: 0-10 min (40-60% B), 10-20 min(60-80% B) at a flow rate of 1.0 mL/min; the column was Welch Ultimate XB-C18 (30 cm × 4.6 mm × 250 mm) with a column temperature of 25 °C and a detection wavelength of 230 nm [1]. The conversion rate, product yield and C_α-C_β bond cleavage selectivity of the lignin model compound were calculated from Equations (S1)–(S3).

$$\text{Conversion of lignin model compounds } (\mathbf{C})=\frac{N_0\text{-}N_t}{N_0}\times 100\% \quad \text{(S1)}$$

$$\text{Product } (\mathbf{X}) \text{ yield}=\frac{N_X}{N_0}\times 100\% \quad \text{(S2)}$$

$$C_\alpha\text{-}C_\beta \text{ bond cleavage selectivity}=\frac{N_2}{C}\times 100\% \quad \text{(S3)}$$

where **C** represents lignin model compounds conversion; N_0 (mmol) is the initial molar amount of the lignin model compound; N_t (mmol) is the molar amount of the lignin model compound in the sample to be measured at the sampling time; N_x (mmol) is the molar amount of the product (**X**) from lignin model compounds; N_2 (mmol) is the molar amount of the benzaldehyde.

Supplementary Figures

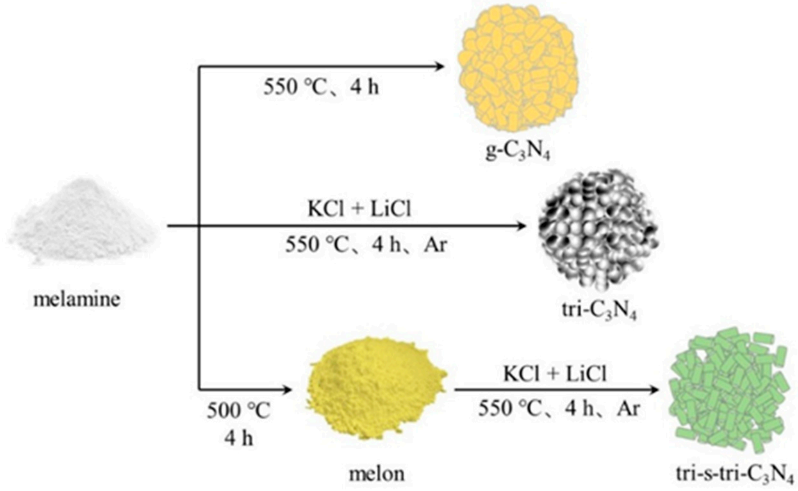

Figure S1. Synthesis roadmap of g-C_3N_4, tri-C_3N_4 and tri-s-tri-C_3N_4.

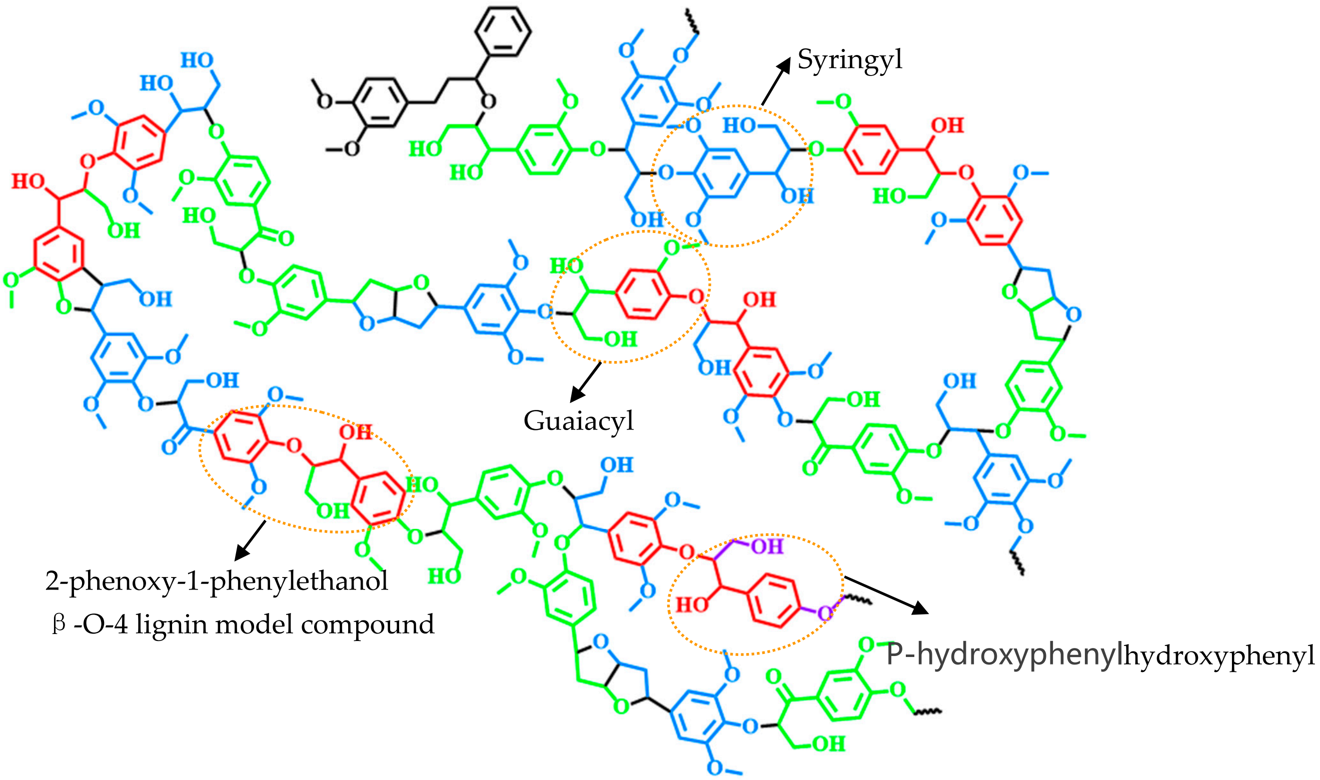

Figure S2. Structure diagram of lignin extracted from *Camellia oleifera* shell [2].

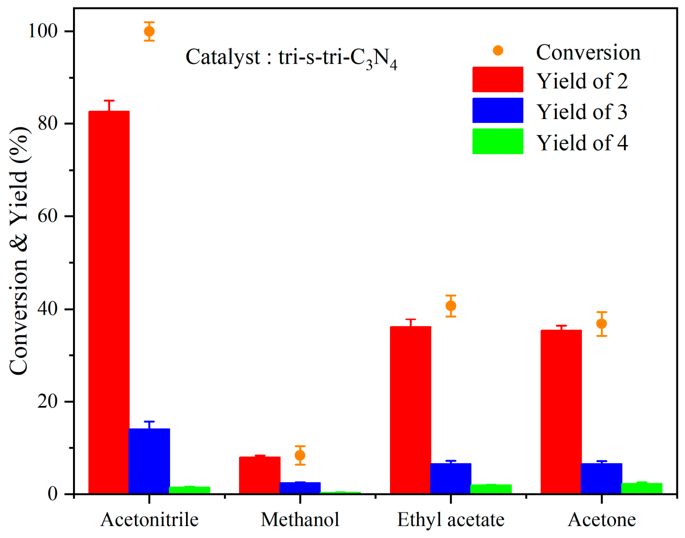

Figure S3. Conversion and product yield of photocatalytic conversion ML of tri-s-tri-C_3N_4 in different organic solvents.

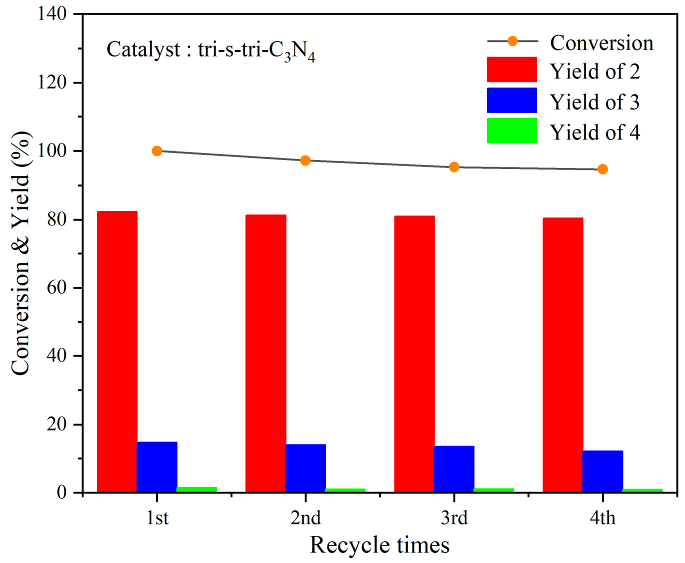

Figure S4. Recycles experiment of tri-s-tri-C_3N_4.

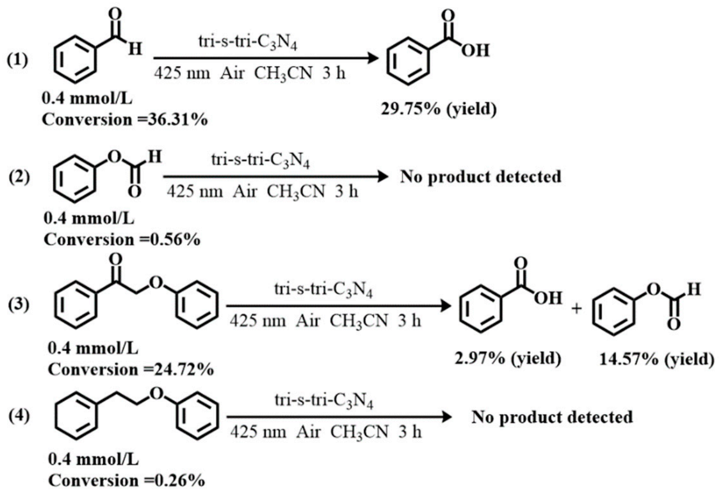

Figure S5. Photocatalytic reaction results of lignin model compound with different structures.

Supplementary Tables

Table S1. The fluorescence attenuation components of fitted g-C_3N_4, tri-C_3N_4 and tri-s-tri-C_3N_4.

Photocatalysts	τ_1 (ns)	Rel %	τ_2 (ns)	Rel %	Ave. τ (ns)
g-C_3N_4	1.0852	89.50	29.6577	10.50	4.0853
tri-C_3N_4	0.8381	48.34	5.3558	51.66	3.1719
tri-s-tri-C_3N_4	0.1924	60.79	3.2833	39.21	1.4043

References

1. Ku, C.H.; Guo, H.Q.; Li, K.X.; Wu, Q.; Yan, L.S. One-step fabrication of mesoporous sulfur-doped carbon nitride for highly selective photocatalytic transformation of native lignin to monophenolic compounds. *Chin. Chem. Lett.* **2023**, 34(1): 107298.
2. Chen, Y.F. Step-by-step hydrothermal preparation of carbon microspheres in *Camellia oleifera* seed shell and study on the mechanism of spherical formation. *Master's thesis, Nanchang Hangkong University*. **2020**, http://doi:10.27233/d.cnki.gnchc.2020.000296.
